# Genetic and functional diversity of ubiquitous DNA viruses in selected Chinese agricultural soils

**DOI:** 10.1038/srep45142

**Published:** 2017-03-22

**Authors:** Li-Li Han, Dan-Ting Yu, Li-Mei Zhang, Ju-Pei Shen, Ji-Zheng He

**Affiliations:** 1 State Key Laboratory of Urban and Regional Ecology, Research Center for Eco-Environmental Sciences, Chinese Academy of Sciences, Beijing 100085, China; 2 University of the Chinese Academy of Sciences, Beijing 100039, China; 3 Faculty of Veterinary and Agricultural Sciences, The University of Melbourne, Parkville, Victoria 3010, Australia

## Abstract

Viral community structures in complex agricultural soils are largely unknown. Electron microscopy and viromic analyses were conducted on six typical Chinese agricultural soil samples. Tailed bacteriophages, spherical and filamentous viral particles were identified by the morphological analysis. Based on the metagenomic analysis, single-stranded DNA viruses represented the largest viral component in most of the soil habitats, while the double-stranded DNA viruses belonging to the *Caudovirales* order were predominanted in Jiangxi-maize soils. The majority of functional genes belonged to the subsystem “phages, prophages, transposable elements, and plasmids”. Non-metric multidimensional analysis of viral community showed that the environment medium type was the most important driving factor for the viral community structure. For the major viral groups detected in all samples (*Microviridae* and *Caudovirales*), the two groups gathered viruses from different sites and similar genetic composition, indicating that viral diversity was high on a local point but relatively limited on a global scale. This is a novel report of viral diversity in Chinese agricultural soils, and the abundance, taxonomic, and functional diversity of viruses that were observed in different types of soils will aid future soil virome studies and enhance our understanding of the ecological functions of soil viruses.

Viruses are the most abundant biological entities and major players across all habitats, reaching 10^31^ virus particles in the Earth^1^. Viruses play very important roles in our environments, such as affecting the community composition and evolution of other microbial groups^2^,^3^,^4^, mediating element biogeochemical cycles in the marine environment^5^. More importantly, vast majority of these viruses in natural environments have no direct consequence to the health of people. Despite the importance of viruses, there is very limited knowledge of the species, abundance, and distribution patterns of viruses in soil ecosystems.

Soil ecologists have long been aware of the need to examine the global biodiversity patterns of soil biota^6^,^7^. To date, there have been many studies of the diversity and biogeography of bacteria, archaea, and fungi in soil^8^,^9^,^10^; however, little work has been focused on soil viral ecology. Recent progress in viral ecological studies were mainly made in more homogeneous marine^5^,^11^,^12^,^13^ and hot spring^14^,^15^ ecosystems, and in soils from harsh environmental ecosystems, such as deserts^16^,^17^,^18^,^19^ and polar regions^20^. The natural viral diversities were deeply studied and found that they were consistently high in local aquatic and soil environments^11^,^12^,^13^,^19^,^20^. However, there was a hypothesis put forward by Breitbart *et al*.^21^ that viral global diversity would be relatively limited as viruses might be moving between environments, which constrains total global viral diversity and provides a conduit for horizontal gene transfer. And then, conserved genes have been used to prove that these phage related sequences are so similar that they must have moved between environments within recent evolutionary time^22^.

Compared with the viral communities in aquatic, soil and other environments, it was observed that the virus types were significantly different from the dominant types identified in marine to soil samples, which suggested that distinct habitat types harbor distinct viral communities^11^,^23^,^24^,^25^. Many studies showed majority of viral particles in soils and sediments belonged to single-stranded DNA (ssDNA) viruses, especially *Microviridae* family^26^,^27^,^28^,^29^. These researches showed that the *Microviridae* sequences comprised 84.6% of whole virus sequences in the Machair soil sample, 50.3% in the brown earth sample and 74% in Izu-Ogasaware Trench deep-sea sediment. The higher presence of ssDNA viruses might have been caused by the use of phi29 multiple displacement amplification (MDA) of the metagenomic DNA before high-throughput sequencing, which has been reported to be biased towards ssDNA^26^,^30^. The diversity of *Microviridae* that are among the smallest of the DNA viruses according to the size of their genomes was always studied by their major capsid proteins (also called CP or VP1) sequences analysis, and four defined subfamilies of *Microviridae* have been divided, including *Gokushovirinae, Pichovirinae, Aravirinae* and *Stokavirinae*^27^.

Up to now, most of what we know about viral ecology in natural systems has been acquired through investigations of aquatic environments. However, in highly heterogeneous soil systems, where microbial populations often form discrete biofilms^19^, viral distribution characteristics could be substantially different from marine systems. Additionally, agricultural activities exert an important influence on the activities and diversity of the soil biota, and different types of agricultural practices and systems affect the soil biota in different ways. Therefore, this study aimed to reveal: (1) the taxonomic and functional diversities of viruses in six typical agricultural soils, (2) the similarities and differences among viromes in this study and other environmental sources, (3) the phylogenetic relationship of major groups existing in these viromes.

## Results

### Abundance and morphology of viruses in the soils

In order to study the morphologic diversity of viruses in the six soils, the shape and size of purified virus particles were examined using transmission electron microscopy (TEM) and characterized into three morphological types: tailed bacteriophage-like particles, spherical particles, and filamentous particles (Fig. S1). The abundance of virus particles identified by epifluorescence microscopy (EFM), and the physical and chemical properties of the soils are shown in Table S1. The abundance of virus particles per gram soil (dry weight) was the highest 1.5 × 10^10^ in the upland chao soil (Shandong-maize), which contained more virus particles than the paddy soils. However, the abundance of virus particles in 1 g (dry weight) of the black paddy soil (Jilin-paddy) was low (5.1 × 10^9^).

### Sequence analysis of six viromes in the soils

After high-throughput sequencing, the metagenomic analysis for the six soil types were computed by MG-RAST and Metavir2; a range of 803,527 to 3,531,567 sequence reads passed quality control by MG-RAST, but less than 30% of the reads that were produced predicted protein coding regions. Detailed information regarding sequencing metadata, assembly metrics, and BLASTx searches are summarized in Table 1. The alpha diversity, which is based on a species-level annotation, summarizes the diversity of organisms in a sample with a single number, and the annotated species richness indicates the number of distinct species annotations in the combined MG-RAST dataset. The species diversity of the six soils differed, but it was interesting to see that the red paddy soil had the highest alpha diversity comprising 329 species, which was approximately 10-fold more diverse than the black paddy soil library (30 species).

Taxonomic hits distribution was based on all of the annotation source databases used by MG-RAST. Pie charts (Fig. S2) illustrate the distribution of taxonomic domains for the annotations. Each slice indicates the percentage of contigs with predicted proteins and ribosomal RNA genes that were annotated to the indicated taxonomic level. Viruses represented the largest fraction (>68%) of the contigs in five soil types, except Jiangxi-maize, in which viruses were the second most common (12.5%) in taxonomic domain.

### Taxonomic diversity of viruses in the soils

**Figure 1:**
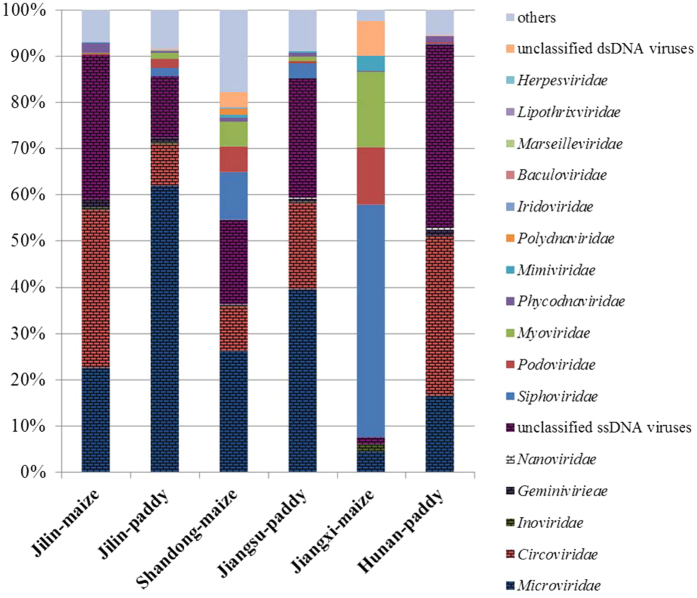
Taxonomic composition of viromes in six typical agricultural soils. Viral taxonomic composition, assessed for all virus-associated contigs at the family level by MetaVir. The fence design represents ssDNA viruses.

The taxonomic distributions of the assignable sequences greatly differed among the six soil types. In the taxonomic composition analysis by Metavir2, five families of ssDNA viruses and 11 families of double-stranded DNA (dsDNA) viruses were distinguished among the six soils (Fig. 1). *Microviridae* were the most abundant in Jilin paddy (61.91%), Shandong-maize (26.15%) and Jiangsu-paddy (39.48%). *Circoviridae* were the most abundant viruses in Jilin-maize (34.28%) and Hunan-paddy (34.61%), while Jiangxi-paddy library comprised 50.36% *Siphoviridae*, which were dominated by the *Caudovirales* order. Additionally, more than 30% of the sequences did not significantly match to any known virus species, which were named viral dark matter. There were also smaller numbers of sequences that matched other ssDNA virus families in the six soil viromes; these included the *Geminiviridae, Nanoviridae*, and even the filamentous bacteriophages belonging to the *Inoviridae*, which were also observed by TEM (Fig. S1).

However, different types of soils had unique distributions of virus species. For example, Eukarya dsDNA viruses *Polydnaviridae, Iridoviridae*, and *Baculoviridae* were mainly found in the Shandong-maize soil, while Archaea viruses *Lipothrixviridae* were only present in Jilin-paddy soil. A small number of reads that displayed the best BLAST hits to disease-causing viruses *Herpesvirales*, which can cause diseases in animals and even in humans, were also found in the red and alkaline upland soil (Jiangxi-maize and Shandong-maize, respectively). Single-stranded DNA plant viuses, *Geminiviridae*, existed in all of the six soils except Jiangxi-maize, and *Inoviridae* resided in all but in Hunan-paddy, while another type of plant viruses *Nanoviridae* were only found in alkaline soils (Shandong-maize and Jiangsu-paddy) and Hunan-paddy soil.

### Structural and functional composition of viromes in the soils

**Figure 2:**
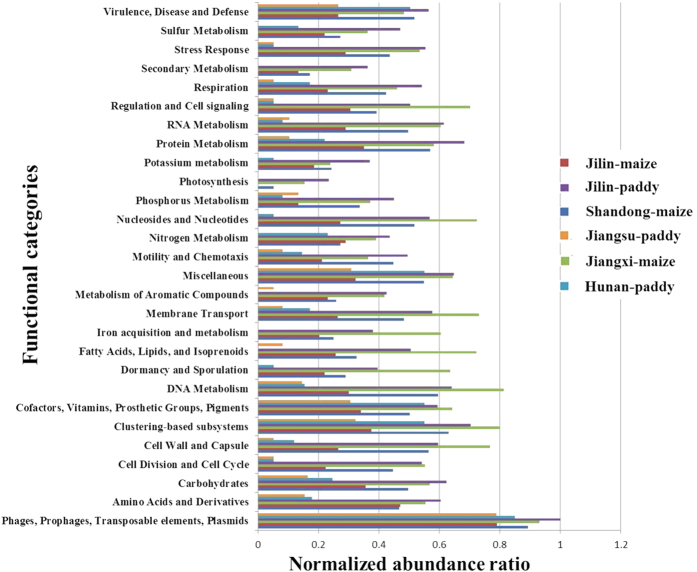
Relative abundance of structural and functional genes based on the predicted ORFs identified by the MG-RAST server.

As the virus genome consists of structural genes and functional genes, we used the MG-RAST to annotate the metabolic sub-systems in the six viromes, and obtained a total of 1,559,051 functional hits through the database. In order to compare the difference among structural and functional genes in the six viromes, each protein was normalized to values between 0 and 1 in Fig. 2, and the original ratio of functional genes were showed in Fig. S3a. A total of 28 functional categories were assigned to the six libraries; the majority (>80%) of which belonged to phages, prophages, transposable elements, and plasmids. Other relatively prominent sub-systems (>1%) included clustering-based sub-systems, DNA metabolism, cell wall and capsule, membrane transport, nucleosides and nucleotides, and fatty acids, lipids, and isoprenoids. Additionally, there were some nitrogen, phosphorus, sulfur, potassium, iron, aromatic compound, RNA, and protein metabolism-related sub-systems. Based on a comparison among the six viromes, we determined that subsystems related to “regulation and cell signaling”, “nucleosides and nucleotides”, “membrane transport”, “iron acquisition and metabolism”, “fatty acid, lipids, and isoprenoids”, “dormancy and sporulation”, “DNA metabolism”, and “cell wall and capsule” were significantly over-represented in Jiangxi-maize compared with the other viromes. The subsystem of phage-specific functional components “phages and prophages” were further investigated and identified as 19 phage-related proteins (Fig. S3b). Phage capsid proteins were the highest content of phage-related functional proteins in all viromes, and the next was r1t-like streptococcal phage proteins. In addition, there were more phage replication, phage packaging machinery, phage integration and excision and *Listeria* phi-A 118-like prophages proteins existing in Jiangxi-maize soil. These functional predictions supported the results of viral taxonomic classifications.

### Comparison of these six viromes with other available viromes

**Figure 3:**
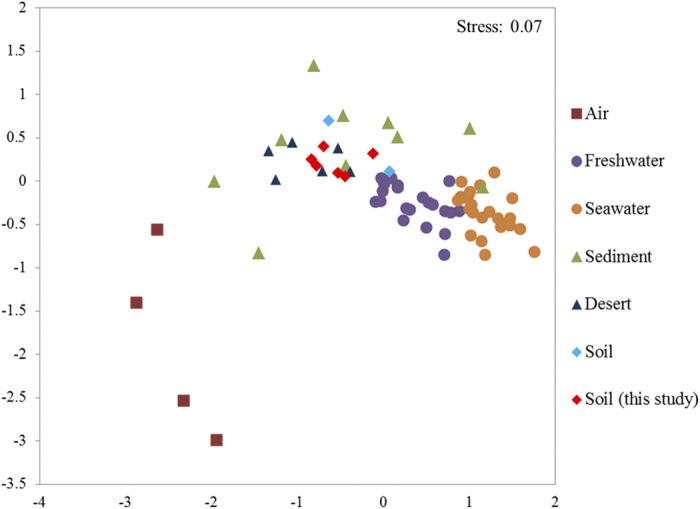
MDS plots of global comparison of viromes from different types of environmental samples using MetaVir2.

In order to compare our viromes with previously published data sets, we selected 74 viromes from different environmental media by NMDS (Non-metric multidimensional Scaling Analysis), including air, freshwater, seawater, desert, sediments and soils (Table S2). Overall, viromes appear to be clustered according to the sample medium types (Fig. 3). For example, all freshwater and all seawater samples grouped together respectively, and the two groups were even closely related with each other. Similarly, all air viromes were clustered together. However, it is very interesting that the viromes from sediment, desert and soil were mixed up together, and their genetic distances seemed far from air and aquatic samples. The six soil viromes in this study were also congregated with some viromes from Antarctic hypolith and open soil, and virome from Namib Desert in Africa.

### Phylogenetic analysis of capsid amino acid sequences of *Microviridae* and terminase amino acid sequences of *Caudovirales*

**Figure 4:**
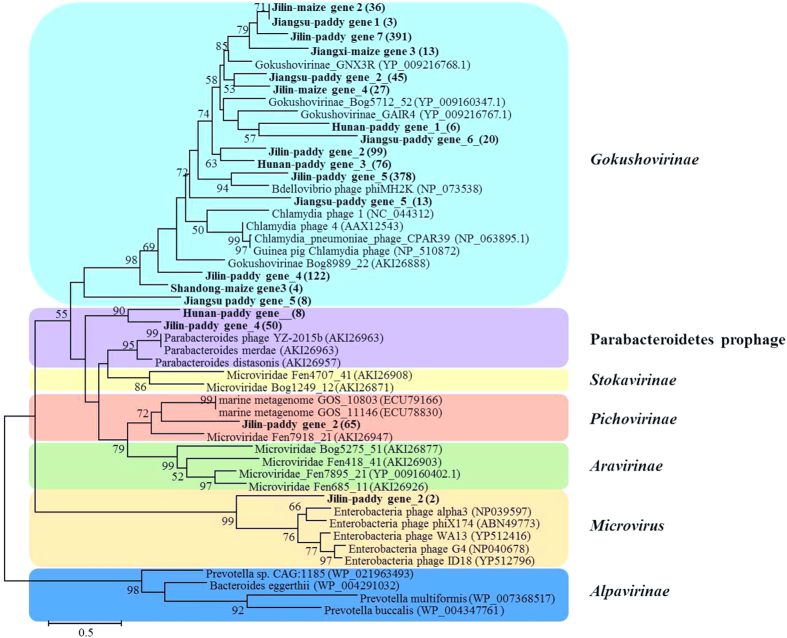
Phylogenetic tree of major capsid protein amino acid sequences of *Microviridae*. The tree was bootstrapped with 1,000 sub-replicates, and confidence levels greater than 50% are indicated at the internodes. The scale bar represents half amino acid substitution per site. The figures in brackets indicate the sequence numbers.

To assess the diversity and genetic distance among the viruses in the six agricultural soils, a phylogenetic tree of *Microviridae* capsid protein, was reconstructed from their contig genes (Fig. 4). A total of 1366 viral capsid protein amino acid sequences (average 500aa) were acquired through clusters of orthologous groups’ functional annotations. Most of the capsid protein genes from the six soil environments clustered together and belonged to the *Gokushovirinae* group^28^, a subfamily of viruses in the *Microviridae* family, which consists of three genera (*Bdellomicrovirus, Chlamydiamicrovirus* and *Spiromicrovirus*). Among the six soils, Jilin-paddy soil had the highest viral diversity, and widely distributed in *Gokushovirinae, Pichovirinae*, Parabacteroidetes prophage and *Microvirus*, according to the classification from Quaiser *et al*.^27^.

**Figure 5:**
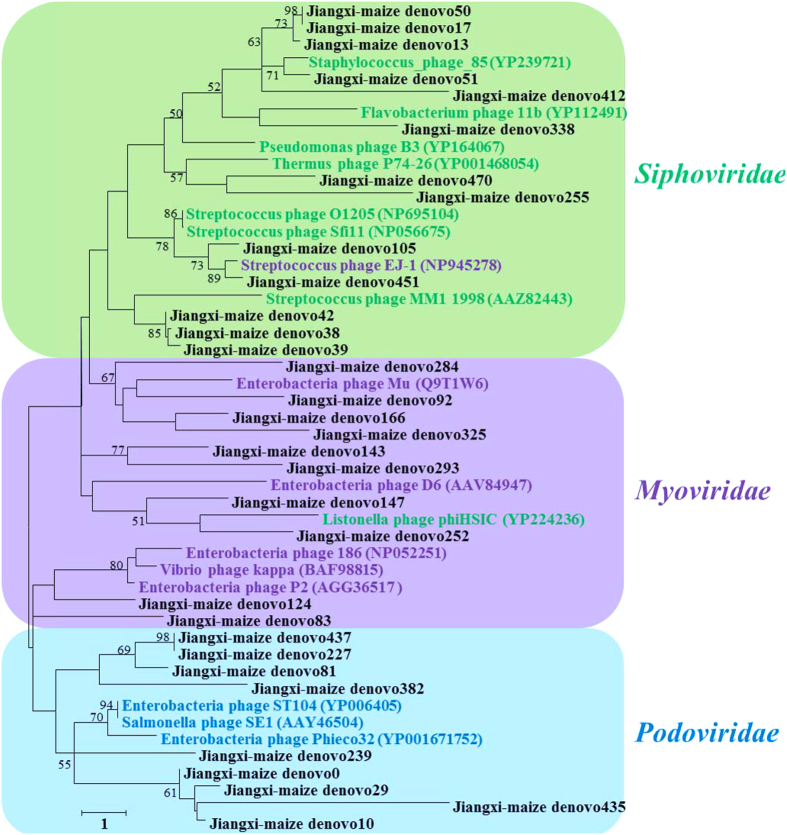
Phylogenetic tree of terminase amino acid sequences of *Caudovirales*. The tree was bootstrapped with 1,000 sub-replicates, and confidence levels greater than 50% are indicated at the internodes. The scale bar represents one amino acid substitutions per site. The purple square indicates *Myoviridae* group, the green square represents *Siphoviridae* group and the blue square is representative of *Podoviridae* group.

As the Jiangxi-maize virome was dominated by bacteriophages, mostly tailed dsDNA phages (*Siphoviridae, Podoviridae* and *Myoviridae*) and small ssDNA *Microviridae* (Fig. 1), phylogenetic analysis of terminase amino acid sequences was used to assess the genetic diversity of *Caudovirales*. We performed phylogenetic tree with amino acid sequences annotated in the Metavir2 analysis as resembling the phage terminase of *Caudovirales* and compared them with the terminase of a wide range of known *Caudovirales* viruses and related metagenomic samples. A total of 32 terminase amino acid sequences (average 350aa) from Jiangxi-maize virome were divided into three main groups related to the *Siphoviridae* group (green in Fig. 5), *Myoviridae* group (purple in Fig. 5) and *Podoviridae* group (blue in Fig. 5), respectively. The results indicated that terminase gene sequences from Jiangxi-maize were diverse and similar to that from other environment samples. Comparing the families within the *Caudovirales, Siphoviridae* represent the major fraction in soil environments, while *Podoviridae* were dominated in seawater and freshwater samples (Table S3).

## Discussion

In our study, the alpha diversities, which are based on the number of species, of the upland black soil and chao soils were higher than those in the paddy soils. An unexpected high species diversity was found in red acidic soils. It is possible that red soil with a coarse texture hinders the spread of microorganisms and inhibits the extraction of virus particles from soils, while the compositions of the microorganisms are relatively simple in alkaline chao soils with high sand contents, which results in low biodiversity. Research by Williamson *et al*.^31^ also found that viral abundance and diversity were greater in a wetland forest soil than in drier agricultural soils. Previous research has shown that the soil bacterial diversity was substantially lower in red soil than chao soil and black soil^32^. Our results illustrate that high viral diversity may be found in systems where the biological diversity of other taxa is low, similar to the case of an Antarctic lake^33^. In that investigation, Lake Limnopolar also harbored greater diversity with viral genotypes than that previously found in viromes from aquatic environments, but had low biological diversity of other taxa.

Sixteen virus families were identified by MG-RAST and Metavir2, and *Microviridae* and *Circoviridae* ssDNA viruses were widespread and the most abundant biotypes in five soil habitats. *Microviridae* is a family of bacteriophages with ssDNA genomes, and it comprises small, spherical viruses that mainly infect enterobacteria, intracellular parasitic bacteria, and *spiroplasma*^34^. A typical ssDNA virus of the *Microviridae* was similarly reported to be the dominant virus in two Scottish soils^28^ and two pelagic sediments^29^. Greater percentages of *Circoviridae* were present in the Hunan-paddy, Jiangsu-paddy and Jilin-maize soils. *Circoviridae* commonly infect birds, mammals, and other eukaryotic hosts^35^, and previous studies showed that the soil moisture content affects soil-inhabiting animals, as evidenced by higher species numbers at higher moisture levels^28^,^36^. It is likely that the higher water content in the Hunan-paddy and Jiangsu-paddy soils increases the number of hosts of *Circoviridae*-related ssDNA viruses, compared with other upland soils. Meanwhile, it should be noted that this study examined the upland soil Jilin-maize, in which the proportion of *Circoviridae* reached 34%. We speculate that this is probably a consequence of applying bio-organic fertilizer composed of mammalian faeces, as many *Circoviridae* existed in the animal faeces from the animal’s intestine.

Our study also expands recent observations of the subfamily *Gokushovirionae* from *Microviridae* family in natural soils, sediments, sewage, and freshwater environments to agricultural soil environments. *Gokushovirionae* are constituted by five genes, and one of the genes coding for capsid protein is much conserved^37^. Therefore, we used the capsid protein gene as a gene marker to investigate the diversity of *Microviridae* family, and our results confirmed that *Microviridae* were diverse and mainly represented by gokushoviruses in agricultural soil. Interestingly, Jilin-paddy soil had higher *Microviridae* viral diversity, including *Gokushovirinae, Pichovirinae*, Parabacteroidetes prophage and *Microvirus*. This will improve our understanding of the significant ecological role of *Microviridae* in different types of environments, which remains an important topic for future study. In addition, the phage terminase of *Caudovirales* has high diversity in Jiangxi-maize soil, and the amino acid sequences of terminase were similar with other phages in marine, freshwater and soil. These results just support the former hypothesis that viral diversity could be high on a local scale but relatively limited globally^21^.

In our study, only 8.5%~24.3% of post quality control sequences were identified as predicted proteins, and most of the sequences were unclear. The rapidly growing database of viral environmental sequences has revealed that most sequences (approximately 90%) do not have homologs in public databases, and are typically labeled as viral dark matter^38^. The same phenomenon occurred that an average of 2/3 of the genes within dsDNA viruses cannot be assigned a biological function or taxonomic affiliation according to a Basic Local Alignment Search Tool homology^19^. This may be one of the reasons why viral species distribution is similar among viromes from different environmental samples. Perhaps more virus species have not been identified clearly. Therefore, virus content and diversity in soils are likely to be underestimated.

A wide range of molecular functions were found in our six viromes, and some of these functions were required for viral reproduction, while others are likely related to metabolic functions. The category of functional genes were strong consistency with that in the viromes of open soil and Hypolith in eastern Antarctic^20^. The original ratio of functional genes (Fig. S3a) displayed a higher amount of sequences of “phages, prophages, transposable elements and plasmids ”. Then the subsystem of phage-specific functional components “phages and prophages” were further expanded and identified as 19 phage-related proteins (Fig. S3b). Among them, phage capsid proteins were ubiquitous and high-content in all viromes, while more phage replication, phage packaging machinery, phage integration and excision and Listeria phi-A 118-like prophages proteins only existed in Jiangxi-maize soil. As viruses are critical for the environment, and they control both bacterial and eukaryotic growth, move genes between cells, and contribute to global geochemical cycles^39^. Meanwhile, viromes appear to act as environment-specific reservoirs of genes with metabolic functions^40^. This suggests that they might play more important and diverse roles than that had previously been suspected.

We compared our six viromes with other selected viromes from different biomes, including air, seawater, freshwater, sediment, desert and soil. Geographical location and environmental physical and chemical properties are known to be the main environmental factors to influence the microbial community structure. However, for all these viral communities and beta diversity analysis, we tend to reach a conclusion that the environmental medium type is the most important driving factor for the viral distribution, of which, physiochemical factors and texture might play more important role than geographical separation. As the virus populations from soil-like media displayed different dynamics from air and aquatic systems. For the air and aquatic systems of homogeneity, the major factors influencing viral community structures were the biological productivity of the system and microbial diversity and abundance^4^,^41^. However, soil-like systems are inhomogeneous and soil particles are semi-discrete^19^, which make against the spread of microorganisms. Thus, we hypothesize that the impact of soil-like media indirectly worked on viral distribution.

The prevalence of *Microviridae* as the dominant taxon was observed in freshwater viromes of Lake Limnopolar, Antarctica^33^ and Lake Bourget in France^42^,^43^,^44^, sediment viromes in Izu-Ogasaware Trench and Shimokita Peninsula^26^ and partial soil samples in Scotland^28^. The higher presence of ssDNA viruses might have been caused by the use of phi29 applied during metagenomic DNA preparation, which have been reproted to result in biased amplification of ssDNA viruses^30^. However, the Jiangxi-maize sample, Namib Hypolith and some other environmental samples^16^ also used MDA and were dominated by dsDNA viruses. Up to now, there was no clear explanation for this characterization and our virus families present in the agricultural soils were also found that similar frequencies in most of the other viromes. In view of the fact that the possible bias of MDA used, as a necessity in viromic studies, we expect that amplication of virus total DNA should be avoided where possible. It would be preferable to focus efforts on reducing the DNA concentration requirements for high through-put sequencing or improving virus extracting concentration to reach the minimum concentrations required for sequencing.

In the present study, diverse ssDNA viruses and a few dsDNA viruses were identified from soils that were collected from six typical Chinese agricultural ecosystems. We extended our knowledge of the viral distribution in several types of soils, particularly for Jiangxi-maize that was dominated by dsDNA viruses and other soils that mainly contained ssDNA viruses, and we showed that the dsDNA virus abundance (as determined by EFM), as well as the community structures, of these viruses were markedly distinct from each other. The different types of viral community structures in Chinese agricultural soils might be affected by land use patterns and soil physicochemical characteristics. Next, the more accurate correlation analysis would be proceeded between viral diversity and soil property using more samples.

In conclusion, our study represents a important survey of viral diversity in six typical agricultural soils in China, and it demonstrated that the taxonomy and functional genes of viruses are diverse, and approximately 30% of viral contigs are uncharacterized. The abundance, taxonomic and functional diversity of viruses that were observed in different types of Chinese agricultural soils will aid future soil virome studies in larger spatial scale and enhance our understanding of the ecological functions of soil viruses. To elucidate the functions of novel genes, further studies of viral diversity in different types of soils are needed.

## Methods

### Soil sample collection

Six soil samples were collected from typical Chinese agricultural areas of the northeast plain (Black soil, Mollisol in the United States Department of Agriculture (USDA) Soil Taxonomy), eastern plain (Chao soil, Ustochrept in the USDA Soil Taxonomy), and southern China (Red soil, Paleudults in the USDA Soil Taxonomy). The Jilin-maize (43°66′N, 126°49′E) and Jilin-paddy (43°66′N, 126°49′E) sites are located in the main agricultural areas of northeast China, where black soil, which is fertile and has a high clay content, is the typical soil. The Shandong-maize (38°11′N, 116°46′E) and Jiangsu-paddy (33°29′N, 120°28′E) sites are located in eastern Chinese agricultural areas, where chao soil, which is alkaline and high in calcium, is the typical soil. The Jiangxi-maize (28°13′N, 116°54′E) and Hunan-paddy (28°55′N, 111°26′E) sites lie in the agricultural region of southern China, where red soil is the typical soil; this soil develops in a warm-wet climate and is usually a low-quality soil that is characterized by a low nutrient content, a low water holding capacity, and a coarse texture. Dryland maize and paddy rice are the main crops in the three major agricultural areas. To study the viral diversity in the six types of soils, approximately 5 kg of soil was collected from each of the six sites in July 2015 and transported at 4 °C back to the laboratory. At each site, we collected three soil sample replicates from three separated 10 m × 10 m plots, by pooling eight upper 10-cm soil cores randomly taken from every plot. For each sample, 1 kg of soil sieved to 1 mm was immediately suspended and filtered, and 500 g of soil samples sieved to 2 mm was stored at 4 °C for physicochemical analyses.

### Virus extraction and purification

The method chosen for virus extraction from the soil samples in this study were mainly refer the previous article of Williamson *et al*.^45^. Five hundred grams of soil per sample was suspended in 1.5 l of a glycine (250 mM, pH = 8.5) solution, shaken for 30 min, and centrifuged at 4000 g for 10 min at 4 °C to precipitate soil particles, and the supernatant was filtered sequentially through 1-mm, 0.45-μm, and 0.20-μm tangential flow filters (GE Healthcare Life Sciences, Pittsburgh, PA, USA). Subsequently, the virus particles in the filtrate were concentrated using 30-kDa centrifugal ultrafiltration tubes (Merck Millipore Ltd., Tullagreen, Ireland) until the final sample volume was less than 1 ml. Finally, viral concentrates were treated with DNaseI (10 units DNaseI/100 ul (Thermo)) and incubated at 37 °C for 1 h to remove free, non-encapsulated DNA. The presence of free and contaminating bacterial DNA was checked by PCR amplification of the 16S rRNA gene with primers 27F/1492R^46^.

### TEM and EFM examination of viral phenotype and abundance

Viral abundances in the soils were estimated by direct counting of virus-like particles (VLPs) under EFM. 100 μl of the final viral concentrates were suspended in 900 μl of sterile deionized water, and vacuum filtered through a 25-mm diameter filters consisting of a 0.02-μm pore size Whatman Anodisc at a pressure less than 62 kPa for each sample. The filters were stained sample side up for 15 min in 100 μl of SYBR Gold working solution (1:10) in the dark. The slides were counted immediately by three-dimensional structured-illumination microscopy (API OMX V3, GE) at the Center for Biological Imaging, Institute of Biophysics, the Chinese Academy of Sciences (CAS). The shape and size of the VLPs were analyzed by TEM (Tecani G20 TWIN, FEI). Concentrated VLPs were collected onto a 400-mesh Cu electron microscopy grid by natural drying that was supported by a carbon-coated Formvar film and incubated at room temperature for 5 min. Then, the grids were stained at room temperature for 2 min with uranyl acetate (2% w/w) and dried on a filter paper. Grids were examined in a Tecnai G2 20 TWIN TEM operated at 200 kV at a magnification of 40,000–85,000 at the Core Facility Center, Wuhan Institute of Virology, CAS.

### Viral DNA extraction and high-throughput sequencing

Viral DNA for high-throughput sequencing was prepared as described by Thurber *et al*.^47^. We used the Power Viral Environmental RNA/DNA Isolation kit (MO BIO Laboratories, Carlsbad, CA, USA) to extract viral DNA. The REPLI-g Mini Kit (for multiple displacement amplification (MDA)) (Qiagen, Hilden, Germany), which utilize Phi29 polymerase, and claim to produce accurate and highly uniform amplification of the entire genome, was used to amplify the total viral DNA to obtain the concentration and quantity needed for high-throughput sequencing. For each sample, 1 μg of DNA was fragmented to approximately 400 bp by Ultrasonic Cell Disruptor (M220, Covaris) and used as a template to create a metagenome library, which was constructed according to the TruSeq™ DNA Sample Prep Kit (Illumina, San Diego, CA, USA) protocol. The libraries were loaded onto flow cell channels for sequencing using an Illumina MiSeq at Shanghai Majorbio Bio-pharm Biotechnology Co., Ltd. (Shanghai, China) to generate 300-bp paired-end reads.

### Analysis of viromes

The original sequences of the six samples were uploaded into Metagenomics Rapid Annotation using Subsystem Technology (MG-RAST) v3.6^48^ to perform quality control, automated annotation, and produce taxonomic and functional assignments. MG-RAST generates taxonomic assignments based on the M5NR database by best hit classification with a maximum E-value of 1e^−5^, a minimum identity of 60%, and a minimum alignment length of 15 amino acids for proteins, and functional categories based on the SEED subsystem database by hierarchical classification using a maximum E-value of 1e^−5^ and a minimum identity of 60%. The barcharts of amino acid sequences for each functional protein were normalized to values between 0 and 1 to allow for comparison of differently sized samples, the normalization was operated by the MG-RAST server and the maximum value (the functional categories of “Phages, Prophages, Transposable elements, Plasmids” in Jilin-paddy) was standarded for 1. The metagenomes are available on the MG-RAST server with the accession numbers 4683369.3, 4683431.3, 4683353.3, 4697387.3, 4683418.3, and 4684108.3 for the Jilin-maize, Jilin-paddy, Shandong-maize, Jiangsu-paddy, Jiangxi-maize, and Hunan-paddy sites, respectively.

The short reads were also assembled using SOAPdenovo^49^ with default parameters. The assembled contigs from six viromes were respectively uploaded to Metavir2^50^ for analyzing viral genomic data. The viral taxonomic composition was computed from a Basic Alignment Search Tool (BLAST) comparison with the RefSeq complete viral genomes protein sequences database from NCBI (release of 2016-01-19) using BLASTp (27.69% of the virome sequences yielded a significant hit (a threshold of 50 on the BLAST bitscore), as displayed by Krona). Comparisons to publicly available viromes from air, freshwater, seawater, sediment, desert and soil (Table S2) were carried out in Metavir by performing tBLASTx virome comparisons^50^. Briefly, a mean score was deduced from each of these tBLASTx pairwise comparisons, and the matrix of these scores (download from Metavir) was then used in an NMDS to plot these viromes by reducing dimension with a Pearson dissimilarity index.

### Phylogenetic analysis

Phylogenetic trees of capsid protein of *Microviridae* and terminase of *Caudovirales* amino acid sequences were reconstructed using MEGA6 software^51^. For the capsid protein tree, the target and reference amino acid sequences were downloaded from Metavir database by the phylogenetic tree computation from viromes. As the number of target sequences was large, the representative amino acid sequences were picked out by clustering at a 60% similarity level. All selected amino acid sequences were aligned by ClustalW, then the gaps and ambiguously aligned positions were deleted. After alignment, the phylogeny tree was constructed using the Jones-Taylor-Thornton (JTT) model, the maximum likelihood method, and bootstrapping using 1000 replicates for each sequence. For the terminase phylogenetic tree, reference amino acid sequences were also downloaded from Metavir database and the examined sequences were all from the contigs of Jiangxi-maize virome. The methods of terminase amino acid sequences alignment and phylogenetic tree construction were as same as capsid protein of *Microviridae*.

## Additional Information

**Accession codes:** The metagenomes are available on the MG-RAST server (http://metagenomics.anl.gov) with the accession numbers 4683369.3, 4683431.3, 4683353.3, 4697387.3, 4683418.3, and 4684108.3 for the Jilin-maize, Jilin-paddy, Shandong-maize, Jiangsu-paddy, Jiangxi-maize, and Hunan-paddy sites, respectively.

**How to cite this article**: Han, L.-L. *et al*. Genetic and functional diversity of ubiquitous DNA viruses in selected Chinese agricultural soils. *Sci. Rep.* **7**, 45142; doi: 10.1038/srep45142 (2017).

**Publisher's note:** Springer Nature remains neutral with regard to jurisdictional claims in published maps and institutional affiliations.

## Supplementary Material

Supplimental Materials

## Figures and Tables

**Table 1:** Details of sequencing of six agricultural soil viromes.

Soil sample	Read data						Contig data	
	Number of reads	Total size (Mb)	Average length (bp)	GC content (%)	α-Diversity (species)	Number of Predicted protein	Number of contigs	Longest contig (bp)
Jilin-maize	803,527	239	297	46 ± 7	48	68,017	14,395	8,021
Jilin-paddy	3,462,453	971	280	48 ± 8	30	797,263	98,888	26,027
Shandong-maize	1,931,704	600	310	44 ± 8	105	468,700	53,164	14,312
Jiangsu-paddy	898,910	289	321	45 ± 7	59	94,601	13,602	15,231
Jiangxi-maize	3,531,567	1,043	295	40 ± 7	191	546,348	119,719	13,238
Hunan-paddy	1,534,532	517	336	48 ± 7	329	170,374	34,882	6,614
