# Supplementary material for: Genetic and functional diversity of ubiquitous DNA viruses in selected Chinese agricultural soils
Source: Sci Rep. 2017 Mar 22;7:45142. doi: 10.1038/srep45142 (PMC5361096; doi:10.1038/srep45142)
Supplement: Supplimental Materials [file srep45142-s1.pdf]

Genetic and functional diversity of ubiquitous DNA viruses in selected Chinese agricultural soils

Authors: Li-Li Han[1,2†], Dan-Ting Yu[1,2†], Li-Mei Zhang[1,2], Ju-Pei Shen[1,2], Ji-Zheng He[1,2,3*]

[1]State Key Laboratory of Urban and Regional Ecology, Research Center for Eco-Environmental Sciences, Chinese Academy of Sciences, Beijing 100085, China

[2]University of the Chinese Academy of Sciences, Beijing 100039, China

[3]Faculty of Veterinary and Agricultural Sciences, The University of Melbourne, Parkville, Victoria 3010, Australia

*Correspondence to: Ji-Zheng He. E-mail: jzhe@rcees.ac.cn

Phone: (+) 86 10 62849788

Supplementary Figure 1. TEM images of viruses extracted from Chinese agricultural soils. Scale bars represent 200 nm.

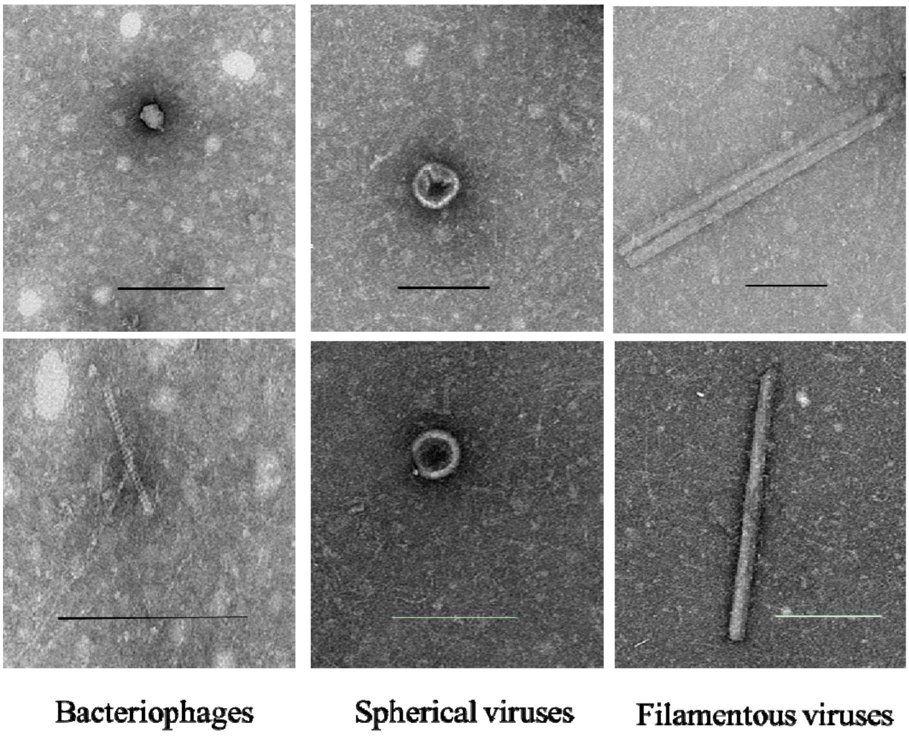

Supplementary Figure 2. Pie charts of taxonomic hits distribution in the six types of soils.

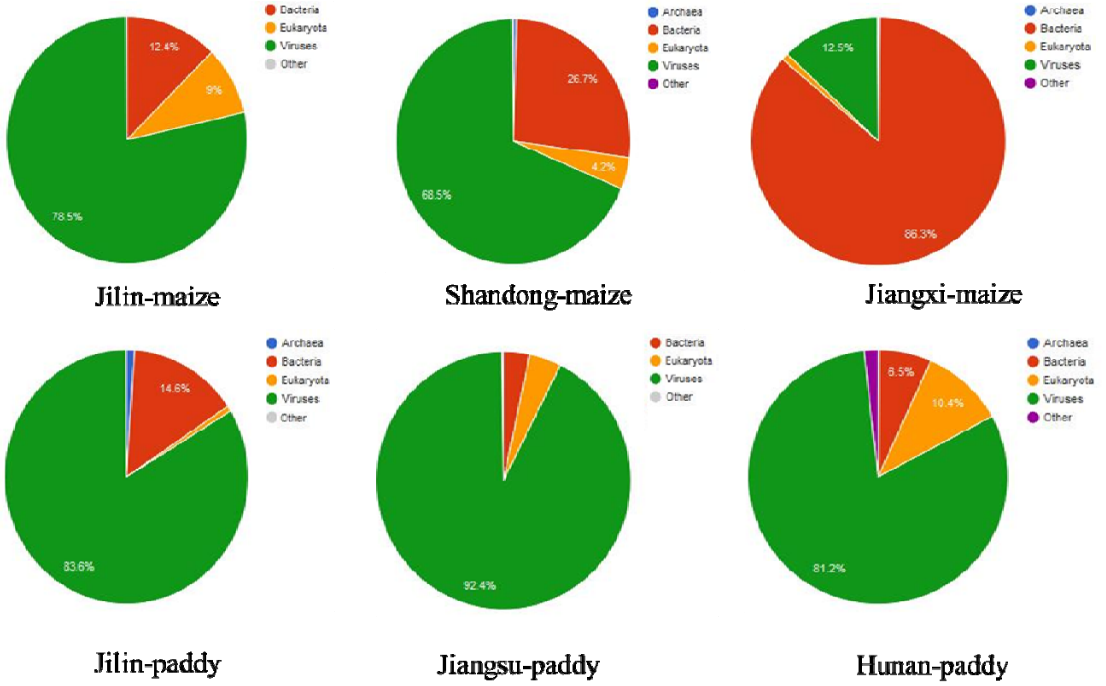

Supplementary Figure 3a. The original ratio of functional genes based on the predicted ORFs identified by the MG-RAST server.

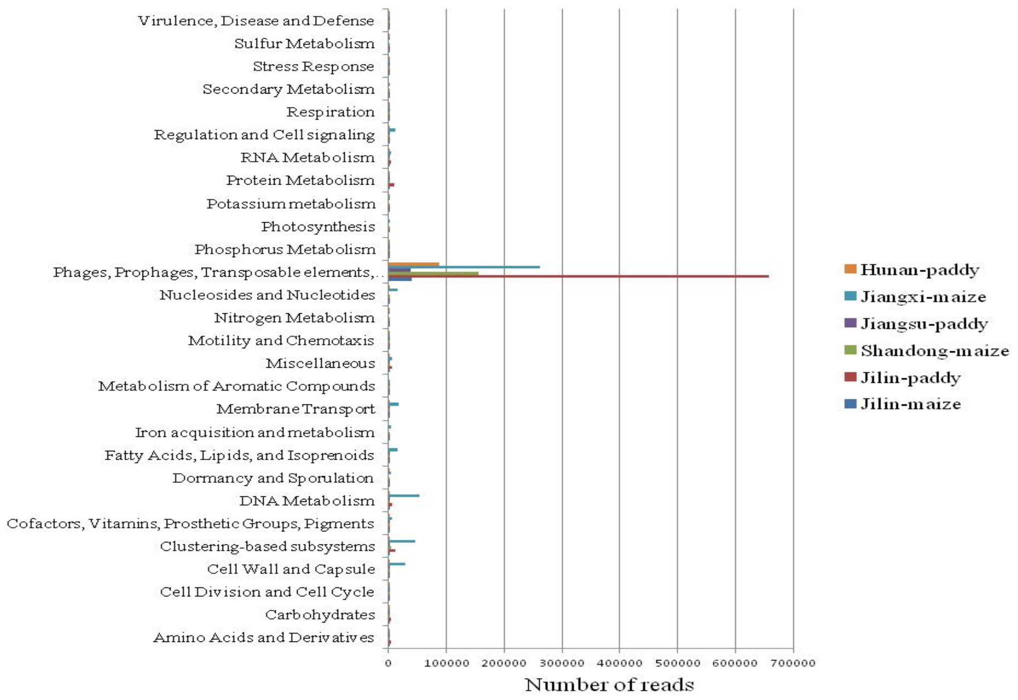

Supplementary Figure 3b. The original ratio of phages and prophages, based on the predicted ORFs identified by the MG-RAST server.

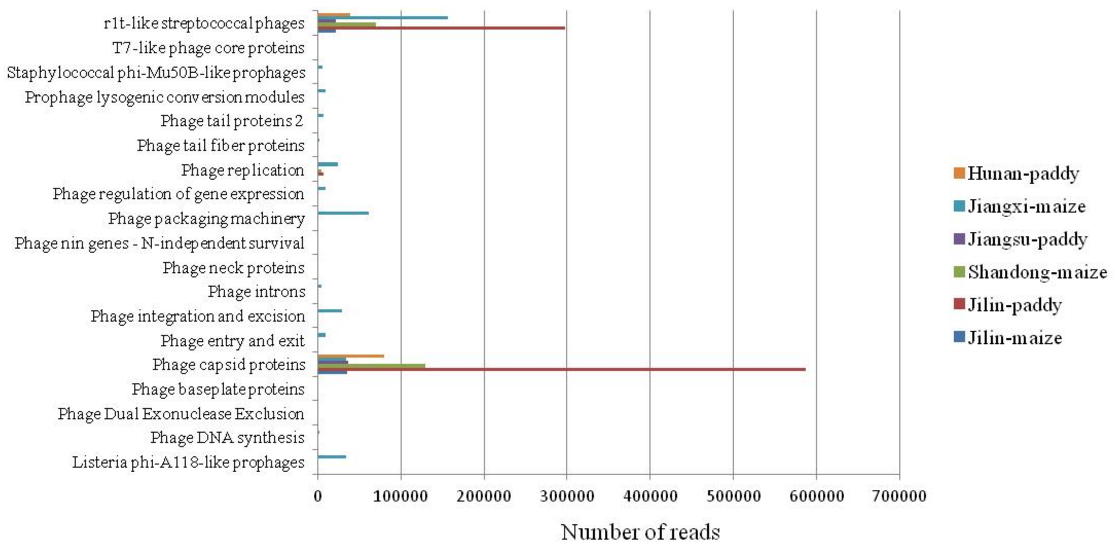

Supplementary Table 1. The physical and chemical properties of six agricultural soil and abundance of virus particles

Soil samples	Agrotype	Crop type	pH	EC(µs/cm)	OM(g/kg)	TN （g/kg）	AP(mg/kg)	AK(mg/kg)	Abundance of viruses($\times 10^{10}$)
Jilin-maize	black soil	maize	4.66	777.00	33.40	1.81	23.45	153.80	1.12±0.15
Jilin-paddy	black soil	paddy	6.08	63.00	43.10	2.24	71.78	125.78	0.51±0.04
Shandong-maize	chao soil	maize	8.29	262.00	25.78	1.48	40.36	150.88	1.50±0.07
Jiangsu-paddy	chao soil	paddy	8.36	244.00	22.28	1.19	48.16	167.08	0.94±0.09
Jiangxi-maize	red soil	maize	4.54	35.00	10.70	0.61	17.19	132.45	0.99±0.03
Hunan-paddy	red soil	paddy	5.42	79.10	39.13	2.08	11.58	81.93	1.01±0.06

pH and EC: determined by pH meter (Professional Meter PP-20, Sartorius, Germany);

OM: Organic Matter, determined using the $K_2Cr_2O_7$ oxidation method;

TN: Total Nitrogen, determined using a Vario EL III analyzer (Elementar Analysensysteme GmbH, Hanau, Germany);

AP: Available Phosphorus, determined using the Olsen method;

AK: Available Potassium, which was extracted with 0.5 M ammonium acetate and determined by an atomic absorption spectrophotometer (ZEEnit700P, Analytik Jena AG, Germany).

The unit on the abundance of virus particles was per gram soil (dry weight).

Supplementary Table 2. List of different types of virome datasets used in this study.

Virome	Geographic zone	NMDS1	NMDS2	NMDS3	Reference
**This study**					
Jiangsu_paddy	Jiangsu, China	-0.52896	0.09267	-0.09896	This study
Jilin_paddy	Jilin, China	-0.779	0.180659	0.108105	
Jilin_maize	Jilin, China	-0.8364	0.253166	-0.09354	
Hunan_paddy	Hunan, China	-0.69296	0.402876	0.118087	
Jiangxi_maize	Jiangxi, China	-0.11735	0.315246	-0.58832	
Shandong_maize	Shandong, China	-0.44532	0.049985	-0.06597	
**Air**					
Air_Residential_district	Seoul, Korea	-2.33007	-2.53175	-0.60253	Whon et al., 2012
Air_Forest	Center Korea	-2.63289	-0.56107	-0.61417	
Air_Industrial_complex	West Korea	-1.9446	-2.98596	-0.28532	
Air_Rainwater	Seoul, Korea	-2.88073	-1.40083	-0.29596	
**Sediments**					
Atl_Vir	Atlantic Ocean	-1.1893	0.473859	-0.50317	Unpublished
Arct_Vir	Arctic Ocean	0.058695	0.675265	0.681169	
Atlantic_Extra	Arctic Ocean	-1.96958	-0.00292	-0.51771	
Arctic_Extra	Atlantic Ocean	-1.45128	-0.82802	-1.50801	
Izu_Ogasawara_Trench	Izu-Ogasawara Trench in the northwest Pacific	-0.47046	0.758765	-0.63684	Yoshida et al., 2013
Mariana_Trench	Mariana Trench in the northwest Pacific	-0.8078	1.334721	-0.15327	
off_Shimokita_Peninsula	off Shimokita Peninsula in the	-0.4343	0.17685	-0.47679	

	northwest Pacific				
Peru_Margin_1mbsf_amplified	Peru Margin	0.165643	0.511027	-1.09796	Unpublished
Peru_Margin_50mbsf_amplified	Peru Margin	1.007283	0.605491	-0.50871	
Brazos_Trinity_8mbsf_	Brazos-Trinity Basin	1.157727	-0.06752	-0.8875	Unpublished
**Desert**					
Antarctic_hypolith_c	Miers Valley, Antarctica	-0.714	0.118421	0.185655	Zablocki et al., 2014
Antarctic_open_soil_c	Miers Valley, Antarctica	-1.05756	0.44933	0.485011	
Antarctic_hypolith_r	Miers Valley, Antarctica	-0.38275	0.111505	-0.2558	
Antarctic_open_soil_r	Miers Valley, Antarctica	-1.33712	0.347804	0.605646	
C60_contigs	Namib Desert	-0.52822	0.384378	0.579659	Adriaenssens et al., 2014
Namib_hypolith_2012_contigs	Namib desert	-1.2495	0.020729	0.31691	
**Soils**					
RFPeru	Manu National Park	-0.63711	0.699085	0.5904	Fierer et al., 2007
BC	Xinjiang, China	0.07086	0.106793	0.004101	Han et al., 2017
**Freshwater**					
AllContigs_VCSEP_2A	Lake Lanier	-0.09607	-0.23995	0.081451	Unpublished
AllContigs_VCJUL_1	Lake Lanier	0.167374	-0.07396	0.042014	
VC_JUL_2	Lake Lanier	0.167374	-0.07396	0.042014	
Lough_Neagh_4pW_contigs	Lough Neagh	-0.03937	-0.23048	0.512145	Unpublished
Antarctic_Lake_Summer	Lake Limnopolar	-0.01406	-0.10981	-0.58286	Lopez-Bueno et al., 2009
FTR_January_2008	Feitsui Reservoir	0.008552	-0.03234	0.070429	Tseng et al., 2012
ElBerbera	ponds of Mauritanian Sahara	-0.02429	0.038181	-0.06857	Fancello et al., 2012
Ilij	ponds of Mauritanian Sahara	0.088408	0.037225	0.10093	

Hamdoun	ponds of Mauritanian Sahara	0.457796	-0.18917	0.243251	
Molomhar	ponds of Mauritanian Sahara	0.161279	-0.047	0.154026	
Jamestown_High_School	Jamestown High School BMP	0.225889	-0.44875	0.299974	Saxton et al., 2016
John_Tyler_199	John Tyler BMP	0.260027	-0.31199	-0.25424	
Crim_Dell	Crim Dell Pond	0.301258	-0.32898	0.223002	
Greensprings	Greensprings Park	0.705603	-0.85216	0.147513	
Matoaka_Open	Williamsburg, VA	0.522313	-0.24163	0.438957	Green et al., 2015
Crim_Dell_Mouth	Williamsburg, VA	0.714895	-0.3454	0.276694	
Lake_Pavin_c	Lake Pavin	0.570696	-0.27	-0.14465	Roux et al., 2012
Lake_Pavin	Lake Pavin	0.49251	-0.53204	-0.22811	
Lake_Bourget	Lake Bourget	0.778089	-0.36312	-0.17476	
Lake_Bourget_c	Lake Bourget	0.877705	-0.34708	-0.26005	
L2010_RNAVirome	Lake Limnopolar, Antarctica	0.760051	0.006777	-0.08784	López-Bueno et., 2015
L2006_RNAVirome	Lake Limnopolar, Antarctica	0.712852	-0.61214	-0.19285	
**Seawater**					
Sargasso_Sea_2005	Sargasso Sea	0.861092	-0.2163	-0.54439	Angly et al., 2015
Dunk_Island_TT_3	Dunk Island	0.896937	-0.17536	0.670613	Hurwitz et al., 2013
Fitzroy_Island_Contigs	Fitzroy Island	0.992523	-0.31525	0.71717	Massey et al., 2013
Dunk_Island_Contigs	Dunk Island	0.928416	-0.18328	0.803672	
Tampa_Bay_induced	Tampa Bay	0.984977	-0.15377	-0.46932	Unpublished
Coral_Atoll_Palmyra	Northern Line Islands	0.905407	-0.0087	-0.63736	Dinsdale et al., 2008
GS112	Indian ocean	1.003071	-0.11725	0.039631	Williamson et al., 2012
GS117	Saint-Anne Island	1.12538	-0.04682	0.134201	
Saanich_200m_r200	Saanich Inlet, Vancouver BC Canad	1.138292	-0.6949	-0.6355	Chow et al., 2015
Saanich_10m_r200	Saanich Inlet, Vancouver BC Canad	1.182757	-0.84652	-0.66964	

2008OMZst3viral200m	Iquique	1.287629	0.104718	-1.83268	Cassman et al., 2012
LA26S	LineP transect, eastern North Pacifi	1.14491	-0.42033	0.44599	Hurwitz et al., 2013
LF26S	LineP transect, eastern North Pacifi	1.226974	-0.35559	0.256938	
M2MS	MBARI transect, intermediate ocea station	1.326953	-0.42837	0.294653	
SFCS	Scripps Pier, San Diego, CA	1.362426	-0.5293	0.625738	
SFDS	Scripps Pier, San Diego, CA	1.391033	-0.50384	0.499515	
LJ26S	LineP transect, eastern North pacifi	1.470894	-0.42904	-0.13045	
SFSS	Scripps Pier, San Diego, CA	1.470964	-0.50847	0.631856	
M5OD	MBARI transect, open ocean statio	1.498676	-0.19471	-0.05854	
M1CS	MBARI transect, coastal station	1.015424	-0.62221	0.67353	
Fitzroy_Island_F1	Fitzroy Island	1.025676	-0.27264	0.539584	
M4OS	MBARI transect, open ocean statio	1.035668	-0.35852	0.523984	
LJ4S	LineP transect, eastern North pacifi	1.588102	-0.55363	0.307956	
LJ12S	LineP transect, eastern North pacifi	1.757716	-0.81118	-0.08438	

Supplementary Table 3. Taxonomic composition of metagenomes in different types of samples by Metavir analysis

Systems	Location	% of total sequences						Reference
		group		order		family		
seawater	Iquique, Chile (10m)	dsDNA	81	*Caudovirales*	66	*Myoviridae*	39	Cassman et al. 2012
	Iquique, Chile (90m)	dsDNA	56	*Caudovirales*	39			
		ssDNA	43	*Circoviridae*	33			
	Iquique, Chile (200m)	dsDNA	89	*Caudovirales*	73	*Myoviridae*	39	
seawater	Northern Line Islands	dsDNA	100	*Caudovirales*	83			Dinsdale et al. 2008
seawater	Indian Ocean	dsDNA	99	*Caudovirales*	79	*Myoviridae*	27	Williamson et al. 2012
						Podoviridae	32	
						Siphoviridae	17	
freshwater	Lake Limnopolar, Antarctica	ssDNA	50	*Microviridae*	40			López-Bueno et al. 2009
freshwater	Lake Bourget in France	dsDNA	67	*Caudovirales*	54	*Myoviridae*	18	Roux et al. 2012
						Podoviridae	15	
						Siphoviridae	19	
		ssDNA	37	*Microviridae*	30			
	Lake Pavin in France	dsDNA	80	*Caudovirales*	66	*Myoviridae*	25	
						Podoviridae	13	
						Siphoviridae	29	
freshwater	Salton Sea (Kent SeaTech) in United States	dsDNA	98	*Caudovirales*	93	*Myoviridae*	19	Rodriguez-Brito et al. 2010
						Podoviridae	46	
						Siphoviridae	27	
freshwater	Jiulong river in China	dsDNA	99	*Caudovirales*	84	*Myoviridae*	26	Cai et al. 2016
						Podoviridae	31	
						Siphoviridae	23	

Sediment	Izu-Ogasaware Trench	ssDNA	81	*Microviridae*	74			
	Mariana Trench	ssDNA	76	*Circoviridae*	36	*Gokushovirinad*	32	Yoshida et al. 2013
	Shimokita Peninsula	ssDNA	97	*Microviridae*	36	*Circoviridae*	50	
Sediment	Arctic Ocean	ssDNA	68	*Inoviridae*	45		29	Dell'Anno et al. 2015
Sediment	Brazos-Trinity Basin in Mexico	dsDNA	95	*Caudovirales*	51	*Phycodnaviridae*	20	Biddle et al. 2011
soil	Antarctic open soil	dsDNA	76	*Caudovirales*	70	*Myoviridae*	25	
						Podoviridae	15	Zablocki et al. 2014
						Siphoviridae	27	
soil	Namib desert	dsDNA	80	*Caudovirales*	75	*Myoviridae*	9	
						Podoviridae	10	Adriaenssens et al. 2014
						Siphoviridae	48	
soil	Coastal soil in Scotland	ssDNA	88	*Microviridae*	84.6			Reavy et al. 2015
	Brown earth in Scotland	ssDNA	71.6	*Microviridae*	50.3			
soil	Jilin-maize	ssDNA	90.2	*Circoviridae*	34.3			
	Jilin-paddy	ssDNA	85.6	*Microviridae*	61.9			
	Shandong-maize	ssDNA	54.6	*Microviridae*	26.2			This study
	Jiangsu-paddy	ssDNA	85	*Microviridae*	39.5			
	Jiangxi-maize	dsDNA	90.1	*Caudovirales*	79.3	*Siphoviridae*	50.4	
	Hunan-paddy	ssDNA	92.5	*Circoviridae*	34.6			

References

Adriaenssens, E.M., Van Zyl, L., De Maayer, P., Rubagotti, E., Rybicki, E., Tuffin, M., et al. (2015): Metagenomic analysis of the viral community in Namib Desert hypoliths. Environ Microbiol 17, 480-495.

Biddle JF, White JR, Teske AP, House CH (2011): Metagenomics of the subsurface Brazos-Trinity Basin (IODP site 1320): comparison with other sediment and pyrosequenced metagenomes. ISME J 5, 1038-1047

Cai L, Zhang R, He Y, Feng X, Jiao N (2016): Metagenomic Analysis of Virioplankton of the Subtropical Jiulong River Estuary, China. Viruses 8, 35

Cassman N, Prieto - Davó A, Walsh K, Silva GG, Angly F, Akhter S, Barott K, Busch J, McDole T, Haggerty JM (2012): Oxygen minimum zones harbour novel viral communities with low diversity. Environ Microbiol 14, 3043-3065

Dell’Anno A, Corinaldesi C, Danovaro R (2015): Virus decomposition provides an important contribution to benthic deep-sea ecosystem functioning. PNAS 112, E2014-E2019

Dinsdale EA, Pantos O, Smriga S, Edwards RA, Angly F, Wegley L, Hatay M, Hall D, Brown E, Haynes M (2008): Microbial ecology of four coral atolls in the Northern Line Islands. PLoS One 3, e1584

López-Bueno A, Tamames J, Velázquez D, Moya A, Quesada A, Alcamí A (2009): High diversity of the viral community from an Antarctic lake. Science 326, 858-861

Reavy B, Swanson MM, Cock PJ, Dawson L, Freitag TE, Singh BK, Torrance L, Mushegian AR, Taliansky M (2015): Distinct Circular Single-Stranded DNA Viruses Exist in Different Soil Types. Appl Environ Microbiol 81, 3934-3945

Rodriguez-Brito B, Li L, Wegley L, Furlan M, Angly F, Breitbart M, Buchanan J, Desnues C, Dinsdale E, Edwards R (2010): Viral and microbial community dynamics in four aquatic environments. ISME J 4, 739-751

Roux S, Enault F, Robin A, Ravet V, Personnic S, Theil S, Colombet J, Sime-Ngando T, Debroas D (2012): Assessing the diversity and specificity of two freshwater viral communities through metagenomics. PLoS One 7, e33641

Whon TW, Kim M-S, Roh SW, Shin N-R, Lee H-W, Bae J-W (2012): Metagenomic characterization of airborne viral DNA diversity in the near-surface atmosphere. J Virol 86, 8221-8231

Williamson SJ, Allen LZ, Lorenzi HA, Fadrosh DW, Brami D, Thiagarajan M, McCrow JP, Tovchigrechko A, Yooseph S, Venter JC (2012): Metagenomic exploration of viruses throughout the Indian Ocean. PLoS One 7, e42047

Yoshida M, Takaki Y, Eitoku M, Nunoura T, Takai K (2013): Metagenomic analysis of viral communities in (hado) pelagic sediments. PLoS One 8, e57271

Zablocki O, van Zyl L, Adriaenssens EM, Rubagotti E, Tuffin M, Cary SC, Cowan D (2014): High-level diversity of tailed Phages, eukaryote-associated viruses, and virophage-like elements in the metaviromes of Antarctic soils. Appl Environ Microbiol 80, 6888-6897
